# Ruthenium(II) carbonyl compounds with the 4′-chloro-2,2′:6′,2′′-terpyridine ligand

**DOI:** 10.1107/S2056989017003917

**Published:** 2017-03-21

**Authors:** Rajendhraprasad Tatikonda, Matti Haukka

**Affiliations:** a University of Jyväskylä, Department of Chemistry, PO Box 35, FI-40014 University of Jyväskylä, Finland

**Keywords:** crystal structure, ruthenium, terpyridine ligand, carbonyl ligand

## Abstract

Two ruthenium carbonyl complexes with the 4′-chloro-2,2′:6′,2′′-terpyridine ligand (tpy-Cl, C_15_H_10_ClN_3_), *i.e.* [RuCl(tpy-Cl)(CO)_2_][RuCl_3_(CO)_3_] (I) [systematic name: *cis*-di­carbonyl­chlorido(4′-chloro-2,2′:6′,2′′-terpyridine-κ^3^ *N*)ruthenium(II) *fac*-tricarbonyltri­chlorido­ruthenate(II)], and [RuCl_2_(tpy-Cl)(CO)_2_] (II) [*cis*-dicarbonyl-*trans*-di­chlorido(4′-chloro-2,2′:6′,2′′-terpyridine-κ^2^ *N* ^1^,*N* ^1′^)ruthenium(II)], were synthesized and characterized by single-crystal X-ray diffraction. The Ru^II^ atoms in both centrosymmetric structures (I) and (II) display similar, slightly distorted octa­hedral coordination spheres. The coordination sphere in the complex cation in compound (I) is defined by three N atoms of the tridentate tpy-Cl ligand, two carbonyl carbon atoms and one chlorido ligand; the charge is balanced by an octa­hedral [Ru(CO)_3_Cl_3_]^−^ counter-anion. In the neutral compound (II), the tpy-Cl ligand coordinates to the metal only through two of its N atoms. The coordination sphere of the Ru^II^ atom is completed by two carbonyl and two chlorido ligands. In the crystal structures of both (I) and (II), weak C—H⋯Cl inter­actions are observed.

## Abstract 2

The Ru^II^ atoms in the crystal structures of two new potential catalyst precursors, [Ru(Tpy-Cl)(CO)_2_Cl][Ru(CO)_3_Cl_3_] and [Ru(Tpy-Cl)(CO)_2_Cl_2_] (Tpy-Cl = 4′-chloro 2,2′:6′,2′′-terpyridine-κ^3^ *N*), exhibit distorted octa­hedral coordination spheres.

## Chemical context   

Ruthenium-carbonyl compounds with polypyridine ligands are known to be active catalysts for several catalytic processes including the reduction of carbon dioxide (Collomb-Dunand-Sauthier *et al.*, 1994 ▸; Chardon-Noblat *et al.*, 2002 ▸; Kuramochi *et al.*, 2015 ▸), water–gas shift reaction (Luukkanen *et al.*, 1999 ▸) and hydro­formyl­ation (Alvila *et al.*, 1994 ▸). Many of these systems are metallopolymers obtained by reducing mononuclear precursors either chemically or electrochemically. The 2,2′-bi­pyridine ligand or its derivatives are the most commonly used ligand systems in these catalysts. It is also reported that possible substituents on polypyridine rings can have a strong impact on the catalytic behaviour of the compounds (Chardon-Noblat *et al.*, 2001 ▸), which could offer a route to tailor the catalytic activity. Compounds with terpyridine and its derivatives as ligands together with carbonyl ligands are less commonly used (Deacon *et al.*, 1984 ▸; Gibson *et al.*, 1997 ▸; Ziessel *et al.*, 2004 ▸), although it has also been shown that these types of compounds can be used to obtain active catalysts. Terpyridines are able to act as strong tridentate ligands because of the arrangement of the pyridine nitro­gen atoms. However, bidentate coordination is also known (Deacon *et al.*, 1984 ▸; Kooijman *et al.*, 2007 ▸; Amoroso *et al.*, 2010 ▸).

In this context we report on the two title compounds, [RuCl(tpy-Cl)(CO)_2_][Ru(CO)_3_Cl_3_] (I) and [RuCl_2_(tpy-Cl)(CO)_2_] (II) with the 4′-chloro-2,2′:6′,2′′-terpyridine ligand (tpy-Cl, C_15_H_10_ClN_3_), which show both types of coordination, *i.e.* tridentate for (I) and bidentate for (II). The title compounds were synthesized by adopting a literature procedure (Homanen *et al.*, 1996 ▸).
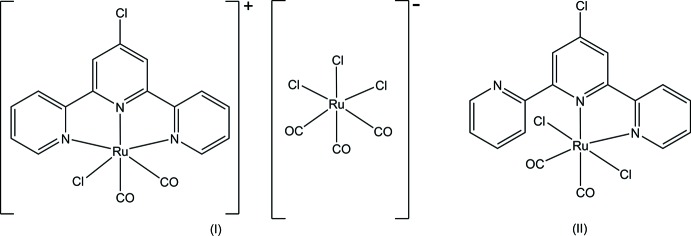


## Structural commentary   

**Figure 1:**
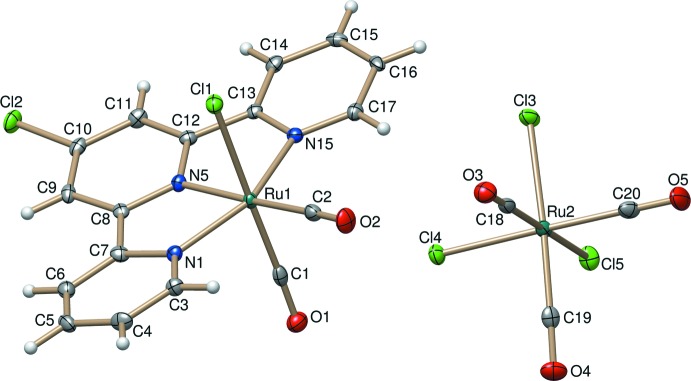
The mol­ecular structures of the cation and anion in compound (I). Displacement ellipsoids are drawn at the 50% probability level.

Compound (I) is a salt and crystallizes in the monoclinic space group *P*2_1_/c with four formula units in the unit cell. The coordination sphere of the Ru^II^ atom in the cation is a slightly distorted octa­hedron. The equatorial positions are occupied by three pyridine N atoms from the Tpy-Cl ligand and by one carbonyl ligand; axial positions are occupied by one chloride and one carbonyl ligand. The charge on the Ru^II^ atom is balanced by an octa­hedrally shaped *fac*-[Ru(CO)_3_Cl_3_]^−^ anion (Fig. 1 ▸). As expected, in the cation the Ru1—N5 bond to the central pyridine ring of the tpy-Cl ligand [2.019 (2) Å] is the shortest of the Ru—N bonds (Gibson *et al.*, 1997 ▸; Ziessel *et al.*, 2004 ▸). The Ru1—N1 [2.097 (2) Å] and Ru1—N15 [2.093 (2) Å] bonds involving the outer pyridine rings are lengthened to relieve strain and to retain a typical terpyridine bite angle of about 79°. Similar structures can be found in other ruthenium(II) complexes containing terpyridine ligands (Gibson *et al.*, 1997 ▸). The Ru1—C2 bond of the equatorial carbonyl group [1.918 (3) Å] is longer than the Ru1—C1 bond [1.893 (3) Å] of the axial carbonyl group, indicating a slightly stronger *trans*-influence caused by the pyridine N atom. The Ru1—Cl1 distance [2.4279 (7) Å] is in the range of typical Ru—Cl bond lengths (Deacon *et al.*, 1984 ▸; Ziessel *et al.*, 2004 ▸). The corres­ponding Ru—Cl bond lengths in the [Ru(CO)_3_Cl_3_]^−^ counter-anion [2.4129 (7)–2.4212 (7) Å] also fall into the typical range of Ru—Cl bonds (Table 1 ▸).

**Figure 2:**
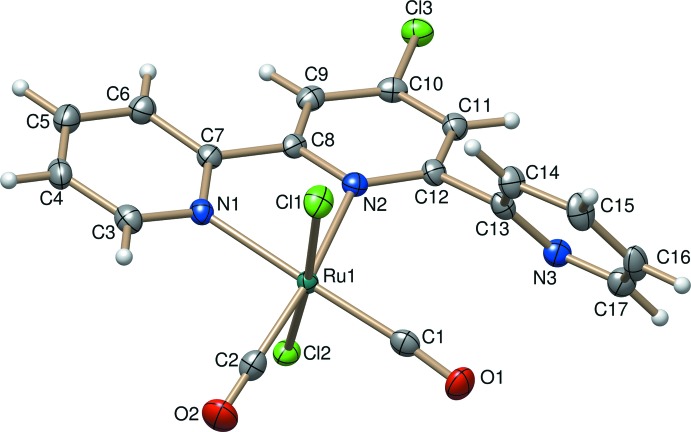
The mol­ecular structure of compound (II). Displacement ellipsoids are drawn at the 50% probability level.

Compound (II) is a neutral complex and crystallizes in the triclinic space group *P* 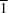 with two formula units. The coordination sphere around the Ru^II^ atom is again a slightly distorted octa­hedron (Fig. 2 ▸). The four equatorial positions are occupied by two N atoms [Ru1—N1 = 2.105 (2) and Ru1—N2 = 2.157 (2) Å] from the Tpy-Cl ligand and by two carbonyl ligands [Ru1—C2 = 1.877 (3); Ru1—C1 = 1.895 (3) Å]. The chlorido ligands [Ru1—Cl1 = 2.3762 (8); Ru1—Cl2 = 2.4098 (7) Å] are placed at axial positions of the mol­ecule. The Ru1—N2 and Ru1—C1 bond lengths are slightly longer than Ru1—N1 and Ru1—C2 bond lengths due to the steric strain generated by the non-coordinating pyridine ring (Table 2 ▸).

The Tpy-Cl ligand in compound (I) is non-planar, despite coordination of all its three N atoms [dihedral angles between the mean planes of the central pyridine ring and the adjacent pyridine rings are 5.70 (8) and 13.28 (7)°]. In compound (II), the ring with the non-coordinating N atom is inclined considerably relative to the coordination plane of the two pyridine rings [dihedral angle 57.71 (9)°].

## Supra­molecular features   

**Figure 3:**
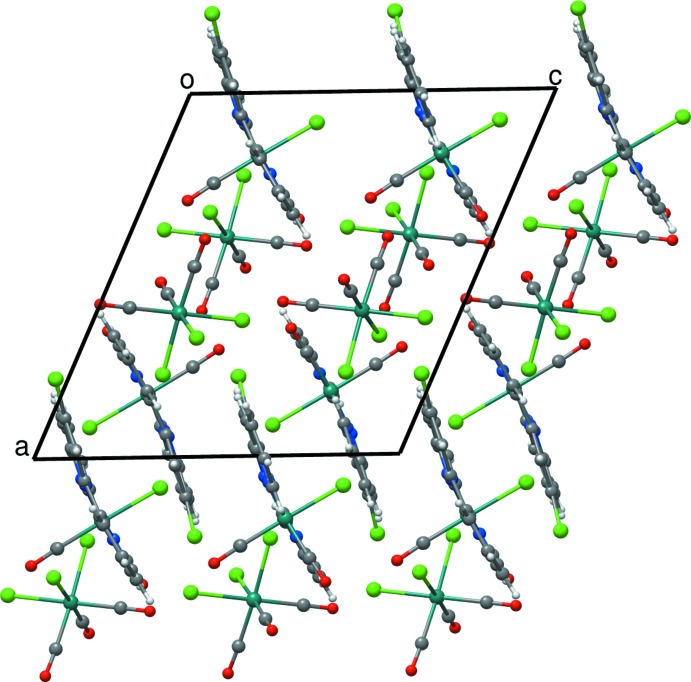
The crystal packing of (I) in a view along the *b* axis.

**Figure 4:**
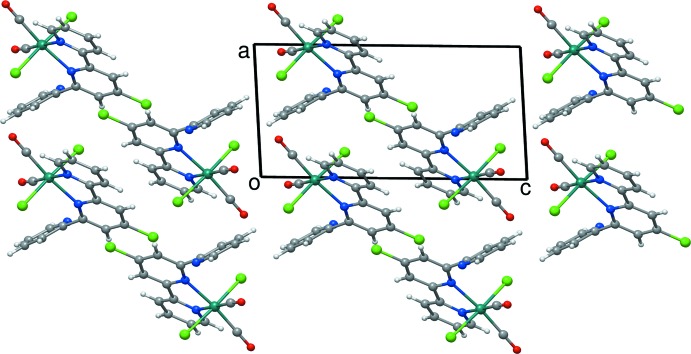
The crystal packing of (II) in a view along the *b* axis.

The packing of mol­ecules (I) and (II) are dominated by van der Waals inter­actions; packing plots are displayed in Fig. 3 ▸ for (I) and Fig. 4 ▸ for (II). Only weak hydrogen bonds and π–π contacts can be found in these structures. In both (I) and (II), some non-conventional hydrogen bonds between the aromatic C—H hydrogen atoms and chlorido ligands of neighboring mol­ecules do exist. The shortest contacts are summarized in Tables 3 ▸ and 4 ▸. In addition to these hydrogen bonds, the aromatic rings in structure (I) are involved in weak face-to-face π–π-inter­actions with considerable offsets. The shortest inter­molecular C—C distances range from 3.23 to 3.50 Å. In (II), an edge-to-face contact exists between C3—H3 and C16 of the neighboring mol­ecule. The distance between H3 and C16 is 2.89 Å and the angle C3—H3⋯C16 amounts to 134°. All inter­actions considered, three-dimensional network structures are obtained both for (I) and (II).

## Synthesis and crystallization   

The title compounds were synthesized using a literature procedure (Homanen *et al.*, 1996 ▸) and both compounds were obtained in a single pot reaction. A solution of [Ru(CO)_3_Cl_2_]_2_ (25.6 mg, 0.05 mmol) in 3 ml of THF was refluxed for 1 h under argon gas. After the reaction time, 26.7 mg (0.1 mmol) of tpy-Cl in 3 ml of THF was added to the above reaction mixture. The resulting mixture was refluxed for another 2 h in air with continuous stirring. During the reaction, the pale yellow solution turned to a reddish solution with a colourless precipitate. The precipitate was collected through centrifugation and the filtrate was evaporated for crystallization. Compound (I) was obtained as a major product originating from the precipitate and compound (II) was collected as a minor product from the filtrate. High-quality crystals of the salt (I) for single-crystal X-ray diffraction were obtained from DMSO solution and those of complex (II) were obtained as brown-coloured crystals from the filtrate.

## Refinement details   

Crystal data, data collection and structure refinement details are summarized in Table 5 ▸. All H atoms were positioned in calculated positions and constrained to ride on their parent atoms, with C—H = 0.95 Å and *U*_iso_ = 1.2*U*_eq_(C). The maximum electron density in complex (I) is located at 0.67 Å from atom C8 and in complex (II) at 1.28 Å from atom N2, respectively. The minimum density in complex (I) is located at 0.77 Å from atom Ru1 and in complex (II) at 0.87 Å from atom Ru1, respectively.

## Supplementary Material

Crystal structure: contains datablock(s) I, II, global. DOI: 10.1107/S2056989017003917/wm5367sup1.cif

CCDC references: 1537190, 1537189

Additional supporting information:  crystallographic information; 3D view; checkCIF report

## Figures and Tables

**Table 1:** Selected bond lengths (Å) for (I)

Ru1—C1	1.893 (3)	Ru2—C20	1.902 (3)
Ru1—C2	1.918 (3)	Ru2—C18	1.914 (3)
Ru1—N5	2.019 (2)	Ru2—Cl4	2.4129 (7)
Ru1—N15	2.093 (2)	Ru2—Cl5	2.4199 (7)
Ru1—N1	2.097 (2)	Ru2—Cl3	2.4212 (7)
Ru1—Cl1	2.4279 (7)	N1—C3	1.336 (3)
Ru2—C19	1.893 (3)		

**Table 2:** Selected bond lengths (Å) for (II)

Ru1—C2	1.877 (3)	Ru1—N2	2.157 (2)
Ru1—C1	1.895 (3)	Ru1—Cl1	2.3762 (8)
Ru1—N1	2.105 (2)	Ru1—Cl2	2.4098 (7)

**Table 3:** Hydrogen-bond geometry (Å, °) for (I)

*D*—H⋯*A*	*D*—H	H⋯*A*	*D*⋯*A*	*D*—H⋯*A*
C11—H11⋯Cl5^i^	0.95	2.76	3.664 (3)	158
C16—H16⋯Cl1^ii^	0.95	2.72	3.515 (3)	142
C5—H5⋯Cl3^iii^	0.95	2.82	3.553 (3)	134

Symmetry codes: (i) 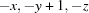; (ii) 
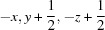
; (iii) 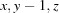.

**Table 4:** Hydrogen-bond geometry (Å, °) for (II)

*D*—H⋯*A*	*D*—H	H⋯*A*	*D*⋯*A*	*D*—H⋯*A*
C9—H9⋯Cl2^i^	0.95	2.77	3.687 (3)	163

Symmetry code: (i) 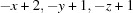.

**Table 5:** Experimental details

	(I)	(II)
Crystal data		
Chemical formula	[RuCl(C_15_H_10_ClN_3_)(CO)_2_][Ru(CO)_3_Cl_3_]	[RuCl_2_(C_15_H_10_ClN_3_(CO)_2_]
*M*_r_	751.70	495.70
Crystal system, space group	Monoclinic, *P*2_1_/*c*	Triclinic, *P* 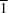
Temperature (K)	123	123
*a*, *b*, *c* (Å)	14.3578 (4), 13.9158 (2), 13.2220 (3)	7.3019 (3), 8.5080 (3), 14.7702 (6)
α, β, γ (°)	90, 114.080 (3), 90	101.287 (3), 91.835 (3), 98.144 (3)
*V* (Å^3^)	2411.86 (11)	889.09 (6)
*Z*	4	2
Radiation type	Mo *K*α	Mo *K*α
μ (mm^−1^)	1.85	1.35
Crystal size (mm)	0.34 × 0.08 × 0.06	0.30 × 0.08 × 0.05

Data collection		
Diffractometer	Agilent SuperNova, Dual, Cu at zero, Atlas	Agilent SuperNova, Dual, Cu at zero, Atlas
Absorption correction	Multi-scan (*CrysAlis PRO*; Agilent, 2013 ▸)	Multi-scan (*CrysAlis PRO*; Agilent, 2013 ▸)
*T*_min_, *T*_max_	0.914, 1.000	0.300, 1.000
No. of measured, independent and observed [*I* > 2σ(*I*)] reflections	11072, 4864, 4264	7508, 3662, 3405
*R*_int_	0.023	0.036
(sin θ/λ)_max_ (Å^−1^)	0.625	0.630

Refinement		
*R*[*F* ^2^ > 2σ(*F* ^2^)], *wR*(*F* ^2^), *S*	0.024, 0.050, 1.06	0.033, 0.088, 1.07
No. of reflections	4864	3662
No. of parameters	316	235
H-atom treatment	H-atom parameters constrained	H-atom parameters constrained
Δρ_max_, Δρ_min_ (e Å^−3^)	0.43, −0.48	0.74, −1.43

Computer programs: *CrysAlis PRO* (Agilent, 2013 ▸), *SUPERFLIP* (Palatinus & Chapuis, 2007 ▸), *SHELXL2014* (Sheldrick, 2015 ▸) and *UCSF Chimera* (Pettersen *et al.*, 2004 ▸).

## supplementary crystallographic information

### Crystal data

**Table:**

[RuCl_2_(C_15_H_10_ClN_3_(CO)_2_]	*Z* = 2
*M**_r_* = 495.70	*F*(000) = 488
Triclinic, *P*1	*D*_x_ = 1.852 Mg m^−^^3^
*a* = 7.3019 (3) Å	Mo *K*α radiation, λ = 0.71073 Å
*b* = 8.5080 (3) Å	Cell parameters from 5231 reflections
*c* = 14.7702 (6) Å	θ = 5.4–76.2°
α = 101.287 (3)°	µ = 1.35 mm^−^^1^
β = 91.835 (3)°	*T* = 123 K
γ = 98.144 (3)°	Plate, brown
*V* = 889.09 (6) Å^3^	0.30 × 0.08 × 0.05 mm

### Data collection

**Table:**

Agilent SuperNova, Dual, Cu at zero, Atlas diffractometer	3662 independent reflections
Radiation source: micro-source	3405 reflections with *I* > 2σ(*I*)
Mirror monochromator	*R*_int_ = 0.036
Detector resolution: 10.3953 pixels mm^-1^	θ_max_ = 26.6°, θ_min_ = 1.4°
φ scans and ω scans with κ offset	*h* = −8→9
Absorption correction: multi-scan (*CrysAlisPro*; Agilent, 2013)	*k* = −7→10
*T*_min_ = 0.300, *T*_max_ = 1.000	*l* = −18→18
7508 measured reflections	

### Refinement

**Table:**

Refinement on *F*^2^	0 restraints
Least-squares matrix: full	Hydrogen site location: inferred from neighbouring sites
*R*[*F*^2^ > 2σ(*F*^2^)] = 0.033	H-atom parameters constrained
*wR*(*F*^2^) = 0.088	*w* = 1/[σ^2^(*F*_o_^2^) + (0.0453*P*)^2^ + 0.5543*P*] where *P* = (*F*_o_^2^ + 2*F*_c_^2^)/3
*S* = 1.07	(Δ/σ)_max_ = 0.001
3662 reflections	Δρ_max_ = 0.74 e Å^−^^3^
235 parameters	Δρ_min_ = −1.43 e Å^−^^3^

### Special details

**Table:**

Geometry. All esds (except the esd in the dihedral angle between two l.s. planes) are estimated using the full covariance matrix. The cell esds are taken into account individually in the estimation of esds in distances, angles and torsion angles; correlations between esds in cell parameters are only used when they are defined by crystal symmetry. An approximate (isotropic) treatment of cell esds is used for estimating esds involving l.s. planes.

### Fractional atomic coordinates and isotropic or equivalent isotropic displacement parameters (Å^2^)

**Table:**

	*x*	*y*	*z*	*U*_iso_*/*U*_eq_	
Ru1	0.97989 (3)	0.30538 (2)	0.20165 (2)	0.01402 (9)	
Cl2	1.19336 (10)	0.26275 (9)	0.31916 (5)	0.02134 (16)	
Cl3	0.53931 (11)	0.29015 (9)	0.58174 (5)	0.02370 (16)	
Cl1	0.76545 (11)	0.37329 (9)	0.09657 (5)	0.02563 (17)	
O1	0.9433 (4)	−0.0448 (3)	0.10113 (17)	0.0312 (5)	
O2	1.2842 (4)	0.3556 (3)	0.07324 (19)	0.0380 (6)	
N2	0.7827 (3)	0.2976 (3)	0.30696 (17)	0.0187 (5)	
N1	1.0061 (3)	0.5492 (3)	0.27105 (17)	0.0183 (5)	
N3	0.6478 (4)	−0.1192 (3)	0.26366 (18)	0.0226 (5)	
C2	1.1703 (5)	0.3368 (4)	0.1217 (2)	0.0242 (6)	
C13	0.6151 (4)	0.0228 (4)	0.2446 (2)	0.0201 (6)	
C12	0.6700 (4)	0.1661 (4)	0.3213 (2)	0.0190 (6)	
C10	0.6327 (4)	0.2955 (4)	0.4765 (2)	0.0197 (6)	
C16	0.5048 (5)	−0.2483 (4)	0.1144 (2)	0.0280 (7)	
H16	0.4700	−0.3452	0.0693	0.034*	
C9	0.7419 (4)	0.4346 (4)	0.4621 (2)	0.0204 (6)	
H9	0.7662	0.5293	0.5096	0.024*	
C11	0.5945 (4)	0.1598 (4)	0.4060 (2)	0.0208 (6)	
H11	0.5186	0.0646	0.4152	0.025*	
C7	0.9276 (4)	0.5750 (3)	0.3533 (2)	0.0190 (6)	
C8	0.8142 (4)	0.4312 (3)	0.3764 (2)	0.0176 (5)	
C17	0.5949 (5)	−0.2517 (4)	0.1970 (2)	0.0271 (7)	
H17	0.6211	−0.3531	0.2076	0.032*	
C1	0.9506 (4)	0.0845 (4)	0.1412 (2)	0.0226 (6)	
C14	0.5230 (4)	0.0391 (4)	0.1639 (2)	0.0236 (6)	
H14	0.5002	0.1420	0.1543	0.028*	
C6	0.9447 (4)	0.7303 (4)	0.4081 (2)	0.0234 (6)	
H6	0.8917	0.7472	0.4665	0.028*	
C15	0.4656 (5)	−0.1013 (4)	0.0977 (2)	0.0281 (7)	
H15	0.4004	−0.0966	0.0418	0.034*	
C3	1.0952 (4)	0.6760 (4)	0.2401 (2)	0.0235 (6)	
H3	1.1461	0.6571	0.1812	0.028*	
C5	1.0398 (5)	0.8593 (4)	0.3764 (2)	0.0261 (6)	
H5	1.0533	0.9657	0.4132	0.031*	
C4	1.1151 (4)	0.8330 (4)	0.2910 (2)	0.0248 (6)	
H4	1.1790	0.9206	0.2678	0.030*	

### Atomic displacement parameters (Å^2^)

**Table:**

	*U*^11^	*U*^22^	*U*^33^	*U*^12^	*U*^13^	*U*^23^
Ru1	0.01375 (13)	0.01423 (13)	0.01338 (13)	0.00090 (9)	0.00123 (8)	0.00191 (9)
Cl2	0.0197 (3)	0.0204 (3)	0.0232 (3)	0.0047 (3)	−0.0033 (3)	0.0023 (3)
Cl3	0.0266 (4)	0.0286 (4)	0.0179 (3)	0.0064 (3)	0.0063 (3)	0.0069 (3)
Cl1	0.0278 (4)	0.0256 (4)	0.0223 (4)	0.0028 (3)	−0.0067 (3)	0.0043 (3)
O1	0.0374 (14)	0.0215 (11)	0.0310 (13)	0.0025 (10)	0.0078 (10)	−0.0031 (10)
O2	0.0325 (14)	0.0397 (14)	0.0417 (15)	0.0039 (11)	0.0167 (12)	0.0068 (12)
N2	0.0167 (11)	0.0194 (12)	0.0205 (12)	0.0036 (9)	0.0012 (9)	0.0043 (9)
N1	0.0172 (12)	0.0179 (11)	0.0194 (12)	0.0015 (9)	−0.0012 (9)	0.0042 (9)
N3	0.0228 (13)	0.0226 (12)	0.0224 (13)	0.0033 (10)	0.0023 (10)	0.0044 (10)
C2	0.0289 (16)	0.0192 (14)	0.0238 (15)	0.0035 (12)	0.0008 (12)	0.0030 (12)
C13	0.0175 (13)	0.0229 (14)	0.0191 (14)	−0.0009 (11)	0.0027 (11)	0.0044 (11)
C12	0.0177 (13)	0.0197 (13)	0.0191 (14)	0.0010 (11)	−0.0004 (11)	0.0043 (11)
C10	0.0205 (14)	0.0256 (14)	0.0141 (13)	0.0069 (11)	0.0014 (10)	0.0043 (11)
C16	0.0315 (17)	0.0237 (15)	0.0224 (15)	−0.0075 (13)	0.0072 (13)	−0.0033 (12)
C9	0.0210 (14)	0.0209 (14)	0.0193 (14)	0.0059 (11)	−0.0018 (11)	0.0028 (11)
C11	0.0188 (14)	0.0207 (14)	0.0225 (14)	0.0012 (11)	−0.0001 (11)	0.0050 (11)
C7	0.0164 (13)	0.0193 (13)	0.0209 (14)	0.0026 (11)	−0.0003 (11)	0.0033 (11)
C8	0.0159 (13)	0.0180 (13)	0.0172 (13)	0.0027 (10)	−0.0032 (10)	0.0004 (10)
C17	0.0279 (16)	0.0234 (15)	0.0296 (17)	0.0032 (13)	0.0070 (13)	0.0043 (13)
C1	0.0208 (14)	0.0271 (16)	0.0208 (14)	0.0037 (12)	0.0052 (11)	0.0069 (12)
C14	0.0248 (15)	0.0238 (14)	0.0207 (15)	−0.0008 (12)	−0.0013 (12)	0.0049 (12)
C6	0.0237 (15)	0.0224 (15)	0.0227 (15)	0.0030 (12)	0.0006 (12)	0.0017 (12)
C15	0.0309 (17)	0.0303 (16)	0.0195 (15)	−0.0057 (14)	−0.0022 (12)	0.0040 (12)
C3	0.0204 (14)	0.0259 (15)	0.0246 (15)	0.0018 (12)	0.0004 (12)	0.0074 (12)
C5	0.0246 (15)	0.0210 (15)	0.0305 (17)	0.0022 (12)	−0.0014 (13)	0.0014 (12)
C4	0.0211 (14)	0.0201 (14)	0.0329 (17)	−0.0008 (12)	−0.0015 (12)	0.0078 (12)

### Geometric parameters (Å, º)

**Table:**

Ru1—C2	1.877 (3)	C16—C17	1.376 (5)
Ru1—C1	1.895 (3)	C16—C15	1.387 (5)
Ru1—N1	2.105 (2)	C16—H16	0.9500
Ru1—N2	2.157 (2)	C9—C8	1.383 (4)
Ru1—Cl1	2.3762 (8)	C9—H9	0.9500
Ru1—Cl2	2.4098 (7)	C11—H11	0.9500
Cl3—C10	1.723 (3)	C7—C6	1.395 (4)
O1—C1	1.135 (4)	C7—C8	1.481 (4)
O2—C2	1.129 (4)	C17—H17	0.9500
N2—C12	1.348 (4)	C14—C15	1.390 (4)
N2—C8	1.361 (4)	C14—H14	0.9500
N1—C3	1.345 (4)	C6—C5	1.384 (4)
N1—C7	1.352 (4)	C6—H6	0.9500
N3—C13	1.344 (4)	C15—H15	0.9500
N3—C17	1.344 (4)	C3—C4	1.384 (4)
C13—C14	1.391 (4)	C3—H3	0.9500
C13—C12	1.490 (4)	C5—C4	1.383 (5)
C12—C11	1.391 (4)	C5—H5	0.9500
C10—C11	1.384 (4)	C4—H4	0.9500
C10—C9	1.387 (4)		
			
C2—Ru1—C1	85.52 (13)	C8—C9—C10	117.9 (3)
C2—Ru1—N1	96.08 (11)	C8—C9—H9	121.0
C1—Ru1—N1	178.40 (10)	C10—C9—H9	121.0
C2—Ru1—N2	171.98 (12)	C10—C11—C12	118.4 (3)
C1—Ru1—N2	101.47 (11)	C10—C11—H11	120.8
N1—Ru1—N2	76.94 (10)	C12—C11—H11	120.8
C2—Ru1—Cl1	90.26 (10)	N1—C7—C6	120.8 (3)
C1—Ru1—Cl1	93.67 (10)	N1—C7—C8	115.5 (3)
N1—Ru1—Cl1	86.24 (7)	C6—C7—C8	123.5 (3)
N2—Ru1—Cl1	93.17 (7)	N2—C8—C9	122.6 (3)
C2—Ru1—Cl2	92.02 (10)	N2—C8—C7	115.3 (3)
C1—Ru1—Cl2	92.10 (10)	C9—C8—C7	122.0 (3)
N1—Ru1—Cl2	87.93 (7)	N3—C17—C16	123.4 (3)
N2—Ru1—Cl2	83.87 (7)	N3—C17—H17	118.3
Cl1—Ru1—Cl2	173.94 (3)	C16—C17—H17	118.3
C12—N2—C8	118.4 (3)	O1—C1—Ru1	175.0 (3)
C12—N2—Ru1	126.9 (2)	C15—C14—C13	117.3 (3)
C8—N2—Ru1	112.57 (19)	C15—C14—H14	121.3
C3—N1—C7	119.5 (3)	C13—C14—H14	121.3
C3—N1—Ru1	125.0 (2)	C5—C6—C7	119.1 (3)
C7—N1—Ru1	115.54 (19)	C5—C6—H6	120.4
C13—N3—C17	116.5 (3)	C7—C6—H6	120.4
O2—C2—Ru1	179.6 (3)	C16—C15—C14	119.1 (3)
N3—C13—C14	124.5 (3)	C16—C15—H15	120.5
N3—C13—C12	114.9 (3)	C14—C15—H15	120.5
C14—C13—C12	120.4 (3)	N1—C3—C4	122.3 (3)
N2—C12—C11	122.1 (3)	N1—C3—H3	118.9
N2—C12—C13	120.7 (3)	C4—C3—H3	118.9
C11—C12—C13	117.1 (3)	C4—C5—C6	119.8 (3)
C11—C10—C9	120.4 (3)	C4—C5—H5	120.1
C11—C10—Cl3	119.0 (2)	C6—C5—H5	120.1
C9—C10—Cl3	120.6 (2)	C5—C4—C3	118.4 (3)
C17—C16—C15	119.1 (3)	C5—C4—H4	120.8
C17—C16—H16	120.5	C3—C4—H4	120.8
C15—C16—H16	120.5		

### Hydrogen-bond geometry (Å, º)

**Table:**

*D*—H···*A*	*D*—H	H···*A*	*D*···*A*	*D*—H···*A*
C9—H9···Cl2^i^	0.95	2.77	3.687 (3)	163

Symmetry code: (i) −*x*+2, −*y*+1, −*z*+1.
